# Exploring the Conformational Equilibrium of Mefenamic Acid Released from Silica Aerogels via NMR Analysis

**DOI:** 10.3390/ijms24086882

**Published:** 2023-04-07

**Authors:** Ilya Khodov, Valentina Sobornova, Valeriya Mulloyarova, Konstantin Belov, Alexey Dyshin, Luís Batista de Carvalho, Peter Tolstoy, Michael Kiselev

**Affiliations:** 1 G.A. Krestov Institute of Solution Chemistry, Russian Academy of Sciences, Ivanovo 153045, Russia; 2 Institute of Chemistry, Saint Petersburg State University, Saint Petersburg 198504, Russia; 3 Molecular Physical-Chemistry R&D Unit, Department of Chemistry, University of Coimbra, 3004-535 Coimbra, Portugal

**Keywords:** aerogel, mefenamic acid, CO_2_ sorption, MAS NMR, ^13^C NMR, T_1_–T_2_ RRCOSY, NOESY

## Abstract

This study examines the influence of mefenamic acid on the physical and chemical properties of silica aerogels, as well as its effect on the sorption characteristics of the composite material. Solid state magic angle spinning nuclear magnetic resonance (MAS NMR) and high-pressure ^13^C NMR kinetic studies were conducted to identify the presence of mefenamic acid and measure the kinetic rates of CO_2_ sorption. Additionally, a high-pressure T_1_–T_2_ relaxation-relaxation correlation spectroscopy (RRCOSY) study was conducted to estimate the relative amount of mefenamic acid in the aerogel’s pores, and a high-pressure nuclear Overhauser effect spectoscopy (NOESY) study was conducted to investigate the conformational preference of mefenamic acid released from the aerogel. The results indicate that mefenamic acid is affected by the chemical environment of the aerogel, altering the ratio of mefenamic acid conformers from 75% to 25% in its absence to 22% to 78% in the presence of aerogel.

## 1. Introduction

Pharmaceutical industries today face the major challenge of delivering poorly soluble drugs and increasing their bioavailability. Two critical aspects of drug development are determining the ideal dosage and creating effective formulations for controlled release [1,2]. Recently, several researchers have proposed various methods based on supercritical fluid technology, such as micronization [3]; the creation of multicomponent crystals [4,5]; microencapsulation [6]; impregnation [7]; adsorbing drugs onto porous carriers [8]; and co-precipitation [9] to address the issue of low dissolution rate. The dissolution rate of drugs is increased by adsorption in an amorphous state [3]. Researchers have noted that the efficiency of such drugs remains unchanged during storage [4,5,6,7]. Singh N. et al. have shown that due to high surface area, silica aerogels (AG) are an effective carrier material for poorly water-soluble drugs [8]. The results show [9] that tetraethyl orthosilicate (TEOS) [10]-doped aerogel had significantly better morphology, structure, and characteristics than the non-TEOS aerogel. It had a higher surface area (264 m^2^/g vs. 220 m^2^/g), larger pore size (8.7 nm vs. 8.3 nm), higher pore volume (0.56 cm^3^/g vs. 0.48 cm^3^/g), and greater porosity (74.9% vs. 69.8%) [9]. These results demonstrate that TEOS-doped aerogel is a better material for applications involving the adsorption of drugs into silica aerogel. Giray S. and Ulker Z. et al. showed that this method of drug delivery reduces the side effects and increases the bioavailability of the drug, especially for poorly soluble drugs [11,12]. One of these compounds is mefenamic acid (MFA) which, due to several restrictions, is not recommended for use as a non-steroidal anti-inflammatory drug [13,14,15]. Therefore, Tan F. et al. searched for ways to improve the form of mefenamic acid in order to repurpose it as a drug and to give developed or forgotten drugs, such as mefenamic acid, a chance to enter a new therapy field [16]. Wang W.H. and co-authors have studied the drug as a treatment for various conditions, including breast cancer, rheumatoid arthritis, and inflammatory bowel disease [17].

Polymorphs of mefenamic acid can be prepared using various techniques, such as solvent evaporation, sublimation, and recrystallization. Additionally, mefenamic acid could be micronized through mechanical milling, supercritical fluid processing, and high-pressure homogenization. Furthermore, spray-drying, melt-extrusion, and supercritical fluid impregnation can be used for loading matrices with mefenamic acid. Each approach has advantages and disadvantages, and the most appropriate method should be chosen based on the desired product characteristics.

Recent studies by Singh N. and Tkalec G. [8,18] have indicated that silica aerogels could be viable for drug delivery systems [19,20]. Researchers investigated the silica aerogels impregnated with the number of active pharmaceutical ingredients (API) by conducting adsorption experiments. The bioavailability of API loaded into silica aerogels was found to be higher than that of pure API [21]. Additionally, silica aerogels have low-cost, non-toxic properties and are non-irritants on the skin and mucous membranes, making them an ideal carrier material for transdermal drug delivery. In this work, high-pressure NMR spectroscopy is proposed for further studies to explore the potential of TEOS-doped silica aerogel as a mefenamic acid delivery system, understanding the process of aerogel impregnation and control of drug form by obtaining information on the aerogel’s preferred conformers and sorption characteristics after impregnation. The used methods include ^13^C, NOESY and RRCOSY NMR in supercritical carbon dioxide and solid state ^29^Si, ^1^H, ^13^C (CP) MAS NMR.

## 2. Results and Discussion

### 2.1. Solid State MAS NMR Analysis of Aerogel Composite Material

The density, Brunauer, Emmett and Teller theory (BET) surface area, porosity, and pore size of commercial aerogel, as summarized in the literature [22,23,24], agree with the values obtained for our samples. Silica aerogel (based on silica precursors with four oxygen neighbors per Si atom) are brittle materials with densities between 0.203 and 0.205 g/cm^3^ and surface areas between 593 and 602 m^2^/g. Pure aerogel has a typical blue tint due to Rayleigh scattering from the mesopores of the silica aerogel. When a porous matrix is impregnated with mefenamic acid, the aerogel changes color to dark yellow, which is caused by a change in mesoporosity (see Figure S2) [25,26].

**Figure 1:**
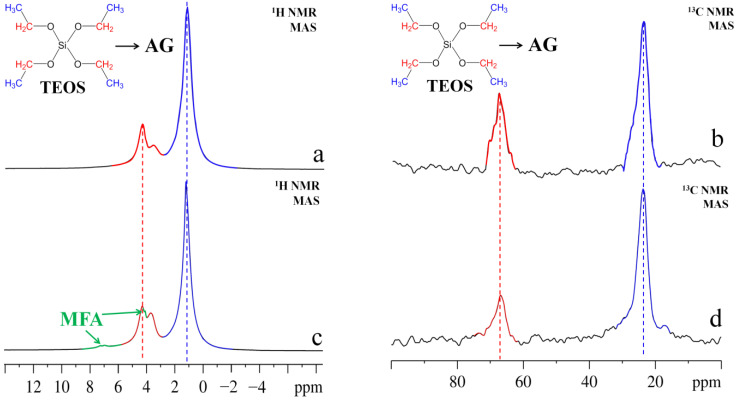
The ^1^H and ^13^C MAS NMR spectra of the original undoped SiO_2_-based aerogel and aerogel doped with mefenamic acid are shown in (**a**,**b**) and (**c**,**d**), respectively, after drying in scCO_2_ medium. The signals corresponding to the hydrogen atoms (**a**) and carbon (**b**) of the CH_2_ groups of the precursor TEOS are marked in red, the signals of the CH_3_ groups of the precursor TEOS are marked in blue, and the signal belonging to the hydrogen atoms of the aromatic and OH/NH fragments of mefenamic acid (MFA) is marked in green.

The ^1^H and ^13^C MAS spectra of aerogels confirmed the structure and provided further insight into silica chemistry. The ^1^H MAS NMR spectrum showed that the signal belonging to the hydrogen atoms of the methyl groups was located at lower frequencies (1.24 ppm) than the signal of the hydrogen atoms of the CH_2_ groups (4.00 ppm). They confirmed the presence of a minor fraction of ethoxy ≡Si(Q_3_)–O–CH_2_–CH_3_ groups for the TEOS samples, as reported in the literature [24,27]. No non-precursor signals were found, indicating the high quality of the aerogel. While the presence of mefenamic acid is not clearly evident from the reported ^1^H NMR spectra (Figure S5), we believe that the signals at ca. 7 ppm and ca. 4 ppm (marked green in Figure 1) stem from mefenamic acid [28]. The presence of mefenamic acid is not evident from the reported ^1^H NMR spectra. The color change shown in Figure S2 is a more clear indication of the acid adsorption and its interaction with the surface silanol groups [29]. It should be noted that detecting minor amounts of mefenamic acid is difficult with NMR because mefenamic groups are characterized by broad ^1^H NMR lines that shift depending on the strength of the intermolecular bonding. The ^29^Si NMR signal analysis can indirectly estimate the mefenamic acid content.

**Figure 2:**
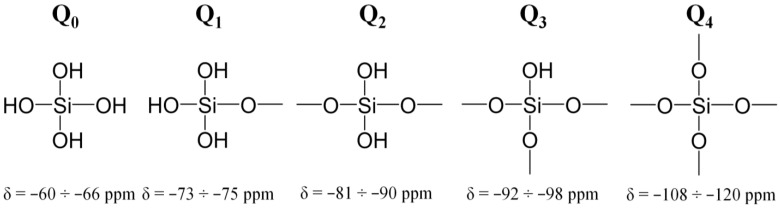
A stereochemical depiction of the Qn silica structural units.

There are five types of Si(O_1/2_)_4_ tetrahedral units (denoted Q_0_, Q_1_, Q_2_, Q_3_, and Q_4_) that can be observed on the ^29^Si NMR spectrum [30,31]. The four different stereochemical units of silicon atoms are the Q_0_ tetrahedron, which has no external connections and is more reactive; the Q_1_ tetrahedron, which has one external connection and is less reactive than the Q_0_; the Q_2_ middle group, which has two external connections; the Q_3_ branching site, which has three external connections; and the Q_4_ cross-linked site, which has four external connections and is the least reactive (see Figure 2) [32,33]. The chemical shifts of the Si atoms in the different units vary due to the different types of chemical environments around the Si atoms and cover the overall range of ca. 60 ppm. Q_0_ has the highest chemical shift of −60 to −66 ppm, while Q_4_ has the lowest chemical shift of −108 to 120 ppm [34]. Meanwhile, the Q_1_, Q_2_, and Q_3_ units have chemical shifts ranging from −73 to −75 ppm, −81 to −90 ppm, and −92 to −98 ppm, respectively [35].

**Figure 3:**
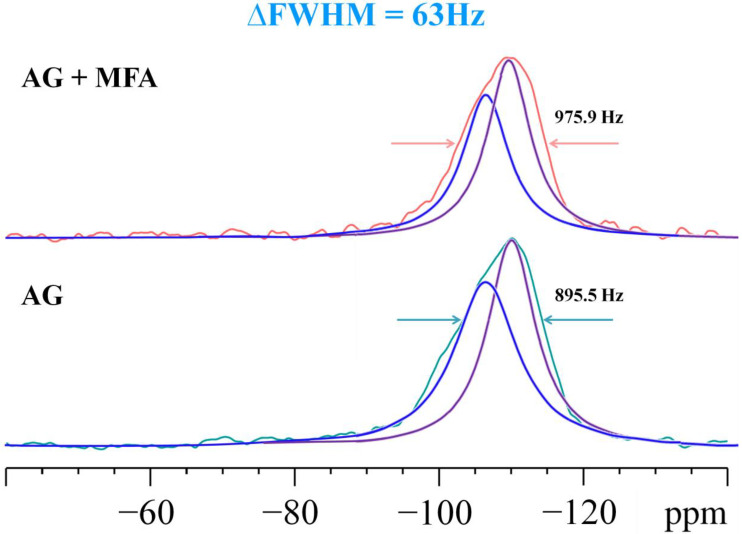
The ^29^Si MAS NMR spectra of pure aerogel (green line) and aerogel with MFA (pink line) and results of pseudo-Voigt profile approximation for deconvolution of signal lines, including Q_3_ (blue lines) and Q_4_ (purple lines) tetrahedral units. ∆FWHM—difference between the widths at half-height signals for doped and pure aerogel.

The ^29^Si MAS NMR spectra of clean, undoped aerogel (see Figure 3) showed the presence of an intense, asymmetrical signal at −108.8 ppm, which can be attributed to Q_4_ cross-linked site [36] silicon atoms forming the aerogel lattice. In the ^29^Si MAS NMR spectra of the aerogel doped with mefenamic acid (see Figure 3), the signal position remained practically unchanged. At the same time, the width increased from ca. 900 Hz (11.3 ppm) to ca. 976 Hz (12.3 ppm). We attempted to observe the change in the coordination states of silica by deconvoluting the signal and analyzing the relative intensities of Q_n_, which signify the states of Si(O_n_) [22,27,37,38]. To obtain this data, we employed the pseudo-Voigt profile approximation to deconvolute the signal lines, with 100% of the Lorentz contribution to the signal shape (see Figure 3).

The profiles of the spectral line approximation by the pseudo-Voigt function showed two pronounced contributions, which, based on the values of chemical shifts, can be attributed to Q_3_ (ac. 100 ppm) and Q_4_ (ac. 110 ppm) [39]. The ratio of Q_4_ to Q_3_ increased from 1.91 for the clean sample to 2.80 for the doped form of AG, suggesting a more cross-linked and, consequently, denser structure with fewer unreacted groups [40]. This is in agreement with the literature data on silylation, which indicates that the increase in the Q_4_/Q_3_ ratio is due to the replacement of the polar and hydrophilic SiOH groups by CH_3_ fragments [41], reducing the number of Q_3_ sites and resulting in a higher Q_4_/Q_3_ ratio [42,43]. The numerical values of Q4/Q3 should be taken with a grain of salt, considering the limited quality of the fit. An alternative interpretation of the ^29^Si NMR test result suggests that the interaction between mefenamic acid and aerogel is not limited to electrostatic forces and involves covalent electron pairs and strong π–π bonds [44]. This could explain the observed signal width and reduced relaxation time [45], which could be attributed to the paramagnetic inclusions in the nearest environment of the aerogel [46,47]. Further research is needed to accurately characterize the effects of these interactions [48,49].

### 2.2. High-Pressure ^13^C NMR Kinetic Study of CO_2_ Sorption

**Figure 4:**
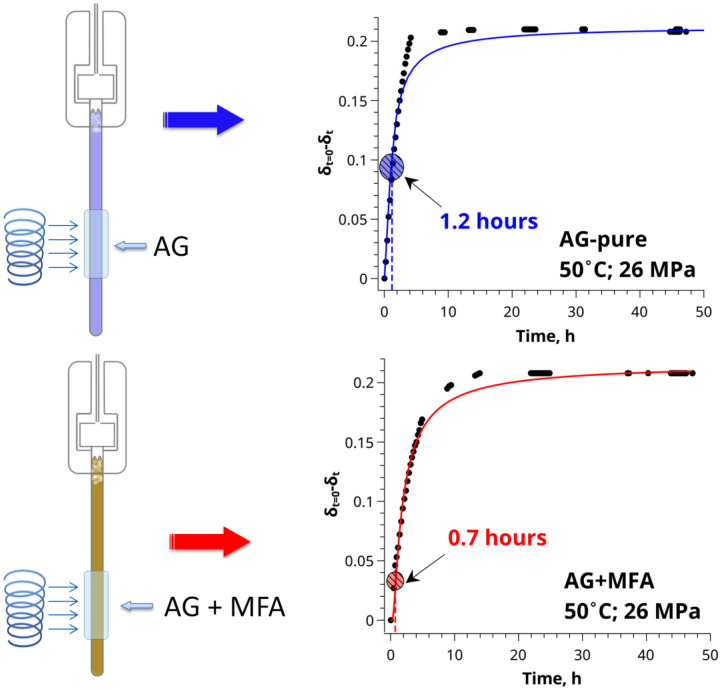
The dependence of the chemical shift parameter of the ^13^C NMR of scCO_2_ signal on time, approximated by a single exponential model, for a system with an initial (blue) and doped (red) aerogel. The red and blue circles indicate the characteristic correlation time for each system.

To identify mefenamic acid in aerogels and understand the doped material physicochemical properties, high-pressure ^13^C NMR experiments were conducted in the aerogel confinement. The ^13^C chemical shift values were obtained and it was found that the observed signals belonged to scCO_2_ carbon atoms. The characteristic kinetic time of scCO_2_ sorption by aerogel was 50 h, where 45 experiments were recorded for both the original aerogel and the one doped with mefenamic acid. The choice of ^13^C NMR as the method for measuring kinetics was due to the rates of CO_2_ sorption being sufficiently high for this parameter. Since the reaction of CO_2_ with the immobilized surface groups is reversible and exothermic, higher temperatures are usually used to take advantage of this. We exploited the fact that that the amount of adsorbed CO_2_ decreases with increasing temperature to reduce the reaction rate and maintain the amount of adsorbed CO_2_ [50,51,52,53]. Anas et al. observed this effect in their study where some aerogels showed significantly higher CO_2_ adsorption capacities at high pressures [54,55]. Therefore, at elevated temperatures and pressures, ^13^C NMR can be used to compare the sorption characteristics of pure aerogel and aerogel doped by mefenamic acid. According to the literature data [56], 26 MPa and 50 °C correspond to the slowest kinetics, allowing for the comparison of the materials with the ^13^C NMR approach. The signals in the ^13^C scCO_2_ NMR spectra were approximated by the Lorentz functions. By analyzing the obtained spectral NMR data, it was possible to identify the characteristic changes in the magnitude of the chemical shift of the observed NMR signal that was used to plot the kinetic curves of the original aerogel (see Figure 4 (blue)) and the one doped with mefenamic acid (see Figure 4 (red)). The kinetic curves were approximated using a single exponential model (Equation (1)), which was previously successfully used for the study of scCO_2_ sorption onto poly(methyl methacrylate) (PMMA) [57].

(1) $$\delta_{t}=\delta_{0}+\delta \exp(-kt)$$

where *δ_t_* is the value of the chemical shift of the CO_2_ signal at time *t*, *δ*_0_ is the value of the chemical shift at the saturation point, *k* is the kinetic rate constant, and *δ* is the multiplier corresponding to the difference between the value of the initial value *δ_t_*_= 0_ (at time *t* = 0) and *δ*_0_.

The application of the proposed mathematical model allowed us to calculate the corresponding correlation times of the process, t_c_ = 1/k, which were 1.2 and 0.7 h for systems with the original and doped aerogel, respectively. These results indicate that the rate of CO_2_ sorption into mefenamic acid with aerogel is 1.7 times slower than the initial rate, likely because some of the aerogel pore surface vacancies are occupied by mefenamic acid, which serves as a limitation to the sorption process. Overall, ^13^C NMR spectroscopy is a suitable method for studying the kinetics of CO_2_ sorption, since it provides direct insight into the chemical environment of the surface groups and can be used to identify the species involved in the reaction. In addition, it can be used to quantify the amount of CO_2_ adsorbed and the rates of CO_2_ uptake. These data provide reliable evidence of the influence of a mefenamic acid small amount on the sorption characteristics of the aerogel composite material, so subsequent experiments were conducted based on the relaxation characteristics by T_1_–T_2_ RRCOSY of the sorption process to estimate the relative amount of mefenamic acid in the aerogel pores. This technique was previously tested on PMMA and demonstrated to be an effective method for determining the sorption characteristics of highly porous materials in a scCO_2_ medium [57].

### 2.3. A High-Pressure T_1_–T_2_ RRCOSY Study

Relaxation–relaxation correlation spectroscopy (RRCOSY) correlates T_1_ with T_2_, offering another method to investigate the pore space in porous media. The RRCOSY technique enables the observation of correlations between T_1_–T_2_ relaxation times through a two-dimensional inversion of the Laplace transform [58]. In RRCOSY, a pair of unique series pulses is used to obtain the 2D relaxation maps of the aerogels samples. Saturation-recovery sequence for the T_1_ and CPMG for the T_2_ relaxation times was used, which was then processed by the two-dimensional inverse Laplace transform (2D-ILT) code created by Venkataraman and co-authors [59]. This technique has been applied to investigate the diffusion process in porous media, for example, in brine-saturated rock [60] and hydrating cement pastes [61,62]. By correlating T_1_ with T_2_, it is possible to calculate the T_1_/T_2_ ratio, which provides insight into the rotational mobility of a molecule and molecular state exchange within a porous system [63]. The intensity of each cross-peak in this spectrum measures the cross-correlation between the T_1_ and T_2_ relaxation times [64]. By analyzing the cross-peak intensities, it is possible to infer the molecular structure of the sample as well as its dynamical properties.

**Figure 5:**
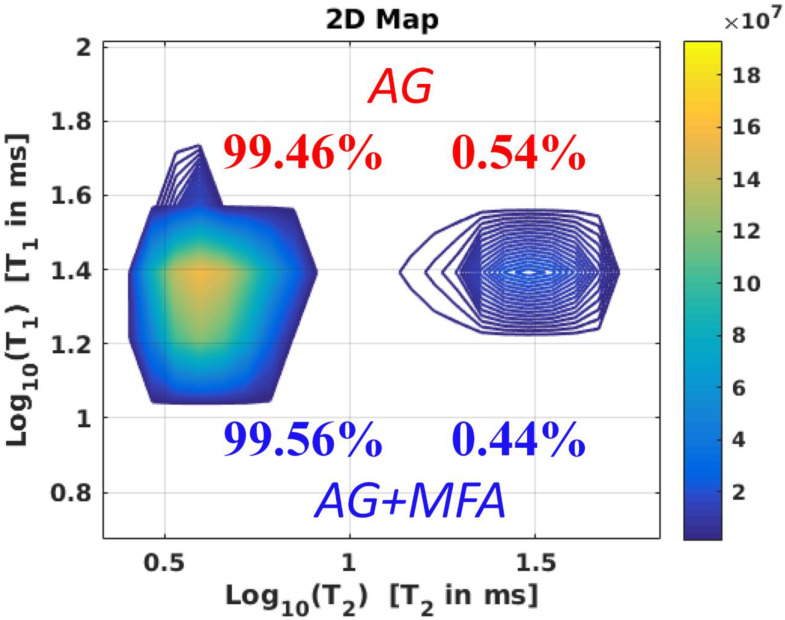
Two-dimensional maps of RRCOSY correlations of carbon dioxide in the original aerogel (red) and the aerogel-doped system (blue) are shown. The logarithms of the relaxation times T_1_ and T_2_ are represented by the ordinate and abscissa, respectively. The maps illustrate a marked disparity in integral intensities between the two systems.

The data obtained from RRCOSY spectra represent populations of specific amplitudes, which are generated due to the various physical regions of mobility of CO_2_ in the material and form different sites. Figure 5 shows two sites on the graph, and two T_2_ values identified but only one T_1_ value can be observed.

The results of the 2D RRCOSY experiment display two prominent peaks: one is situated at the most extended values of T_1_ and T_2_. At the same time, the second is an exchange peak located at the extended values of T_1_ and short T_2_. The exchange process averages T_1_ relaxation processes, making it challenging to isolate internal T_1_ values; hence, only one T_1_ value is observed. Nevertheless, internal T_2_ values can still be obtained using direct data analysis. One of the populations, which appears as a non-diagonal peak, is located within the parity line T_1_ = T_2_, where data representing sites of free solvent scCO_2_ may be found. According to the literature, the farther the population is from the parity line, the more limited the rotational mobility of molecules is [57,60]. The closer the sites are to the parity line, the freer the rotational mobility. Therefore, there are two specific sites in pure and composite aerogel containing mefenamic acid—these sites are physically responsible for binding free CO_2_ molecules and are impregnated into the aerogel pores. Numerical integration of these sites results in a slight increase in the concentration of CO_2_ in the aerogel pores, as expected, compared to the free ones. The integral intensities of the 2D RRCOSY spectra for pure and composite aerogels containing mefenamic acid were compared, showing slight differences in the integral intensities of the signals. The normalized integral intensity values for pure aerogel ranged from 0.54% to 99.46%, while the integral intensity values for composite aerogel containing mefenamic acid ranged from 0.44% to 99.56%. This indicates differences in the chemical environment of the two aerogels, likely due to mefenamic acid in the composite aerogel. These additional results suggest that the presence of mefenamic acid affects the chemical environment of the aerogel, which is reflected by the slight changes in the integral intensities of the 2D RRCOSY spectra.

### 2.4. High-Pressure NOESY Study of the Conformational Preference of Mefenamic Acid Release

One of the critical aspects of this research is investigating how a small dosage of mefenamic acid in an aerogel can influence the conformational equilibrium upon release. This inquiry is significant when constructing drug delivery systems. By understanding how the drug responds to different environmental conditions, researchers can tailor the aerogel to deliver the drug effectively. Additionally, these data can be used to develop a model that predicts the conformational behavior of the drug in different scenarios. This model can then be used to optimize existing drug delivery systems and design new ones.

The determination of interproton distances from NOE data, which we have previously discussed, is based on comparing relative NOE intensities for pairs of nuclei in NOESY experiments [65,66,67]. Suppose we assume that the sample being studied is in the extreme narrowing regime and that the initial rate approximation [68] is valid. In that case, the normalized NOE intensity between two nuclei *I* and *S*, *η_IS_*, is proportional to the experimental cross-relaxation rate, *σ_IS_*, between them and the mixing time, *τ_m_*, of the experiment (Equation (2)).

Furthermore, the cross-relaxation rate, *σ_IS_*, between spins *I* and *S* is proportional to the internuclear distance between them (*r_IS_*^−6^), as demonstrated by Equation (3). This technique can be used to determine the interproton distances of small molecules. For further details, refer to the provided references [69,70,71,72,73,74].

(2) $$\eta_{IS}=\sigma_{IS}\tau_{m}$$

(3) $$\sigma_{IS}\sim r_{IS}^{-6}$$

Assuming that the values of ω (Larmor frequency), *τ_c_* (rotational correlation time), and γ (magnetogyric ratio) remain constant for each nuclear pair in a given 2D NMR experiment, the ratio of intensities of a pair of NOE signals, *η_I_*_(1)*S*(1)_:*η_I_*_(2)*S*(2)_, can be proportional to the ratio of their internuclear distances (Equation (4)) for isotropic intramolecular motion. As such, by comparing *η_I_*_(1)*S*(1)_ and *η_I_*_(2)*S*(2)_ within the same 2D NOESY experiment [75,76], we only need to know the reference distance of one of the nuclear pairs, depending on the conformer, e.g., *r_I_*_(1)*S*(1)_, in order to calculate the experimental distance of the other, *r_I_*_(2)*S*(2)_.

(4) $$\frac{\eta_{I\left(1\right)S(1)}}{\eta_{I\left(2\right)S(2)}}={\left(\frac{r_{I\left(1\right)S(1)}}{r_{I\left(2\right)S(2)}}\right)}^{6}$$

Determining internuclear distances becomes more challenging when analyzing a flexible molecular system with multiple conformations. A general approach to the treatment of small molecules using NOE experiments for both conformational and population analysis is outlined in the reference [71]. The main idea is that, in a narrow approximation, we can identify groups of conformers that may be realized by quantum chemical calculations based on the values of distances as the main criterion. Each distance is proportional to the cross-relaxation rate, and the experimentally observed rate is a weighted average of all possible conformers (Equation (5)).

(5) $$\sigma =\sum_{i}\sigma_{i}x_{i}$$

**Figure 6:**
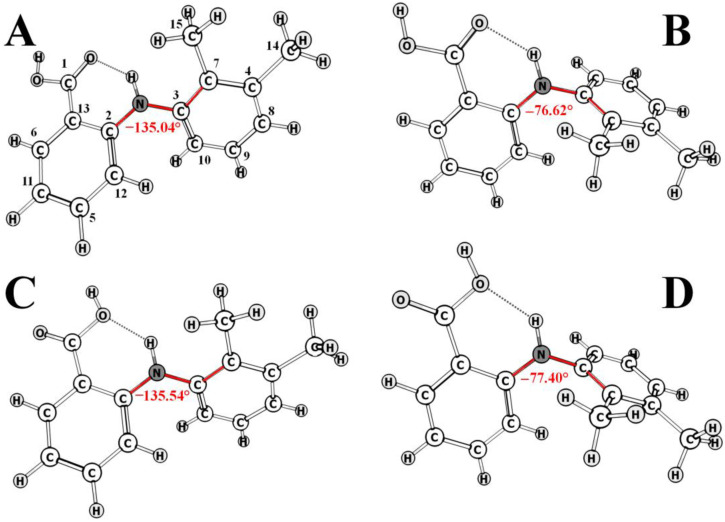
Conformers of the MFA molecule (**A**–**D**) are indicated by the number of carbon atoms and their associated hydrogen atoms; the numbering of atoms remains the same for the remaining conformers. The red color indicates the C2-N(H)-C3-C7 dihedral angle, and its value results vary in different conformations of MFA.

For example, in the case of mefenamic, when analyzing the mobility of benzene rings and the internuclear distance responsible for it, we are dealing with two groups of conformers, A + B and C + D, as described in our previous work [77]. The results of quantum chemical calculations are provided in detail in the literature. The range of MFA conformers (A, B, C, and D) is attributed to the alteration in the values of the C2-N(H)-C3-C7 angle from −135° (A and C) to −77° (B and D) (Figure 6). As demonstrated in [78], these conformers are present in various polymorphic forms of MFA. By comparing the relaxation rates of the two groups, we can determine the relative populations of each conformer. Additionally, suppose the experimentally observed rates differ significantly from the theoretically calculated ones. In that case, other conformers likely exist, and the molecule structure should be further analyzed.

For the accurate calculation of the cross-relaxation rate of each conformer, it is essential to accurately average the intermolecular lability within the groups under consideration for which the distance is determined. Previously, the Tropp model was the most effective in considering intramolecular mobility. As discussed in our paper, we have suggested a semi-empirical coefficient for spherical harmonics [79]. Using this method [80], we can accurately calculate the average cross-relaxation rate of all conformers in a system.

Sternberg and Witter [81] showed that using an incorrect averaging model could lead to misinterpretations of NOESY data, thus hampering the accurate evaluation of the conformational preferences of small molecules. This finding warns against the indiscriminate application of averaging models in NOESY data interpretation and suggests that more sophisticated models should be employed.

On the one hand, in the framework of this study, it was interesting to observe how the release of mefenamic acid from aerogel could affect its conformational preferences in CO_2_ bulk solution by NOESY. The accurate determination of the populations of API molecules has always been limited by the low concentration of API in CO_2_ and the low accuracy of the 2D NOESY-derived restraints used, thus making it impossible to determine conformer populations that fit the observed 2D NOESY sensitivity.

On the other hand, with the high accuracy provided by the NOESY distance analysis, we have recently identified and quantified a previously unrecognized conformer of fenamates by measuring NOESY-derived interproton distances across the phenyl ring of fenamates in CO_2_ with 2% DMSO [77]. This interring distance was observed to be ~3.3 Å for mefenamic and tolfenamic acids and ~3.9 Å for flufenamic acid under the same condition. This ratio of conformers relative to the interring distance is 70:30 for mefenamic acid, and 20:80 for flufenamic acid, respectively [82,83]. This difference in distances was attributed to the conformational lability of flufenamic acid due to the influence of the methyl group [84]. We sought to investigate the conformer populations of mefenamic acid where the intensities of conformational exchange cause its release from aerogel and, hence, any difference in conformational preference for this API.

Within the scope of this work, 2D NOESY spectra of mefenamic acid were recorded in the presence of a doped aerogel (see Figure S3). To achieve this, aerogel samples and mefenamic acid dissolved in DMSO-d6 were introduced into a high-pressure NMR cell. Then, CO_2_ was supplied to the cell and spectra were recorded at 45 °C and 9 MPa. The data interpretation was based on previously obtained results in [77] and Figure S4. The selection of the state parameters was based on the solubility of DMSO-d6 in scCO2, analyzed according to the literature data [85].

**Figure 7:**
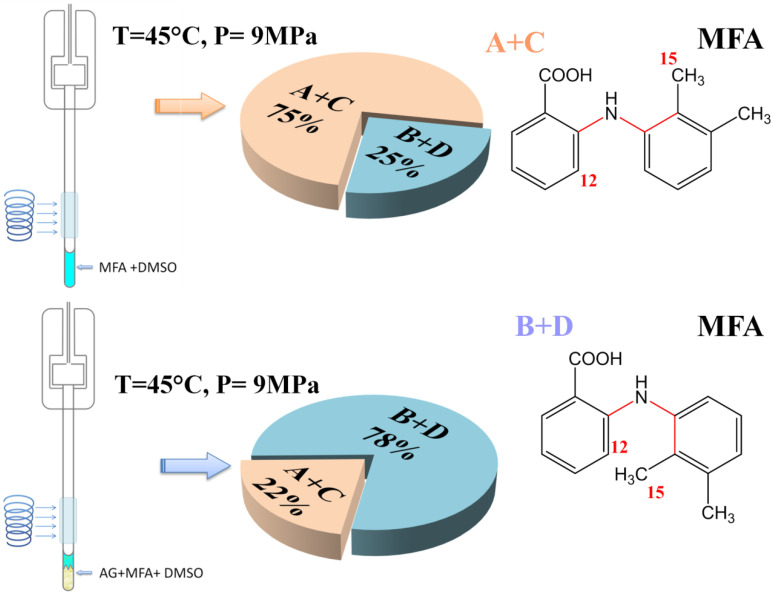
A schematic representation of the cell is shown on the left, with MFA solution in DMSO-d6 (86 μL) depicted in blue and MFA-doped aerogel in yellow. The center displays the distribution of mefenamic acid conformers in scCO2 by adding 2 mol.% DMSO-d6 and in scCO2 + DMSO-d6 in the presence of doped aerogel, calculated at 45 °C and 9 MPa based on the observed conformationally determined H12–H15 distance. On the right, the structures of the predominant MFA conformers are shown, with the numbers indicating the hydrogen atoms of the conformationally defined distance—H12–H15. The red color indicates the chemical bonds that form the dihedral angle, which alteration leads to the different conformations of the MFA molecules.

As previously mentioned, the B3LYP/6-311 + G(2d,p) [77] conformational search of mefenamic acid unsurprisingly yielded two non-degenerate low-energy conformers, the phenyl lability conformation (Figure 7). Each of the conformers, A + B and C + D, differ only by the orientation of the phenyl group. In each case, the A + B conformer (characterized by inter-ring distances of H9/10-H11/12—3.12 Å) is one in which the methyl protons of the benzene ring are positioned on the same side of the carboxyl-substituted benzene ring as the carbonyl group. The C + D conformer (characterized by inter-ring distances of H9/10-H11/12—4.62 Å) is one in which the methyl protons of the benzene ring are positioned on the opposite side the carboxyl-substituted benzene ring to the carbonyl group.

When aerogel was added, the experimental values of cross-relaxation rates changed from *σ_ij_* (1.09 × 10^−2^ s^−1^) and *σ*_0_ (3.75 × 10^−2^ s^−1^) without aerogel to *σ_ij_* (0.63 × 10^−2^ s^−1^) and *σ*_0_ (1.74 × 10^−2^ s^−1^) with aerogel. The addition of aerogel significantly increased the cross-relaxation rates. This is because aerogel is a porous material that can increase the surface area available for the diffusion of molecules, allowing for a more efficient exchange of energy between molecules. H6–H11/12 was chosen as the reference distance, with a value of 2.76 Å (r_0_^calc^) for all conformers. This enabled us to estimate the distances and determine the proportions of conformers. The experimental value for the distance between H12 and H15 was 3.25 Å for mefenamic acid inside the aerogel matrix and 3.81 Å for bulk mefenamic acid. These changes in internuclear distances resulted in the alteration of the ratio of conformers from 22% to 78% in the presence of aerogel from 75% to 25% in its absence (Figure 7).

In conclusion, this study has demonstrated that the NOESY analysis of mefenamic acid can accurately determine both the relative populations and internuclear distances of the different conformers of mefenamic acid in CO_2_ solution. We found that adding aerogel to the solution causes a shift in the conformer populations of mefenamic acid from A + B to C + D, increasing the cross-relaxation rate. The findings of this study provide further insight into the structural and conformational features of mefenamic acid, as well as a general approach to the conformational analysis of small molecules using 2D NOESY data. Thus, determining the conformational compositions of mefenamic acid in CO_2_ is essential for a better understanding of the impregnation process. The results of this study also demonstrated the considerable influence of aerogel on the conformation of the doped molecules, suggesting that aerogel can be used to modulate the conformational state of molecules, which could be beneficial for a variety of applications.

## 3. Materials and Methods

Compounds produced by Sigma-Aldrich were used for NMR experiments, including mefenamic acid (CAS: 61-68-7; MFA, Sigma Aldrich (Darmstadt, Germany), with a purity of at least 99.99% (*wt*/*wt*)) and DMSO-d_6_ (CAS: 61-68-7; 99.9 atom percent D). Carbon dioxide was purchased from Linde Group Company (Balashiha, Russia) (GOST 8050-85, with a purity of 99.995% CO_2_ and less than 0.001% H_2_O). TEOS-doped silica aerogel samples were provided by the Boreskov Institute of Catalysis SB RAS (Novosibirsk, Russia). The synthesis and characterization of these samples has been described in detail in previous works [86,87].

Mefenamic acid was impregnated into the aerogel using a supercritical method in a 100 cm^3^ autoclave at a pressure of 350 bar at temperature 100 °C for ten days. A system for separating the initial components was installed to prevent direct contact between the aerogel sample and mefenamic acid throughout the entire process of impregnation. This adsorption method was unique in that the interaction of the dopant with the aerogel was carried out without physical contact with the components, only through the phase of the scCO_2_ solution, in which mefenamic acid is dissolved. Schematically, a part of the installation (autoclave) is shown in Figure S1. A detailed description of the autoclave, separator, and impregnation technique was previously presented by us [88].

Solid-state ^1^H, ^13^C, and ^29^Si NMR spectra were recorded at 298 K with magic angle spinning (MAS) at 10 kHz using a Bruker 400WB Avance III spectrometer (400.23 MHz for ^1^H, 100.64 MHz for ^13^C, 79.51 MHz for ^29^Si) equipped with a 4.0 mm MAS dual probe. The number of scans was set to 8 (^1^H), 1088 (^13^C), 6184 (^29^Si, doped silica aerogel), and 1568 (^29^Si, pure silica aerogel). The relaxation delay was 30 s (^1^H), 2 s (^13^C), and 10 s (^29^Si).

Such a relatively low value of the relaxation delay for 29Si was previously shown to be sufficient for aerogel systems [89], including those with TEOS [90]. We note, however, that for other silica-based materials, relaxation delays exceeding hundreds of seconds [91,92] and sometimes even hundreds of minutes [93] are far more common.

^13^C and RRCOSY NMR spectra were recorded using a Bruker Avance III 500 spectrometer with a Bruker 5 mm TBI probe. Experiments were conducted on a pure silica aerogel sample and a composite silica aerogel containing mefenamic acid. The characterization of the spatial structure of mefenamic acid and the calculation of the proportions of conformers in the presence of an aerogel doped with mefenamic acid was determined using the data obtained on the same equipment.

**Figure 8:**
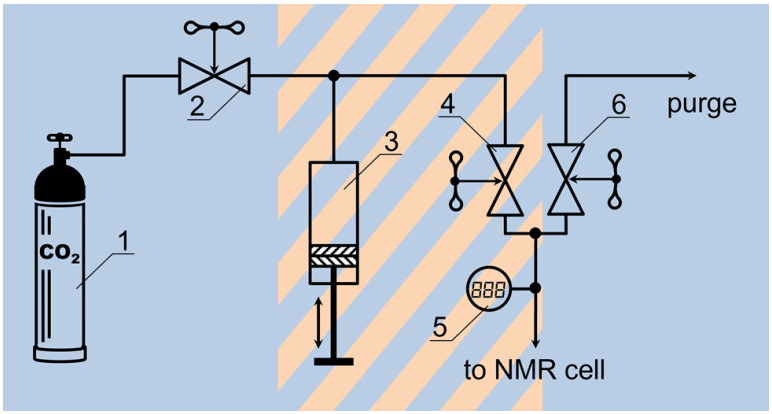
Scheme of the setup for high-pressure NMR experiments at supercritical parameters of state. It consists of a cylinder with carbon dioxide (1), needle valves (2, 4, and 6), a syringe pump (3), and an electronic pressure sensor (5).

An approach based on the spectroscopy of the nuclear Overhauser effect, developed in our previous works [69,70,73], was used to solve this problem. Experiments at supercritical parameters of the state of the solvent (CO_2_) were conducted using the unique scientific installation “Fluid-spectrum” of the G.A. Krestov Institute of Solutions Chemistry of RAS (see Figure 8).

A tube filled up to the flange with aerogel powder was used to study the kinetics of CO_2_ sorption by aerogel. A system of capillaries and needle valves was used to feed carbon dioxide from a cylinder into the tube (see Figure 8). The pressure inside the cell was maintained at 26 MPa using a hand press. The temperature was maintained at 50 °C by an air thermostat, which included a BVT-2000 attachment with an additional cooling module—a Bruker BCU unit with an air flow of 535 L/h. Previously, temperature calibration was performed using a standard K-type thermocouple. The selection of experimental parameters was based on the literature data [94], which indicated that an increase in pressure leads to an increase in both the sorption kinetics and the concentration of the sorbed material. Therefore, the optimal pressure value for this modification of the NMR cell was chosen.

Given the low solubility of mefenamic acid in scCO_2_, sample preparation required a particular approach, using a small amount of DMSO-d_6_ to improve solubility. This technique had been described in previous works [69,74,77] and it has been shown [83] that DMSO-d_6_ in the amount of 2 mol. % does not significantly affect the conformational preferences of fenamates. Sample preparation for determining the conformational preferences of mefenamic acid in a supercritical fluid (scCO_2_) medium involved placing a small amount of crushed silica aerogel in a sapphire ampoule below the coil registration zone. Then, 86 μL of a solution of mefenamic acid in DMSO-d_6_ was added to the ampoule, followed by filling the remaining volume of the ampoule with carbon dioxide from a gas cylinder (see Figure 8). The selection of state parameters (45 °C and 9 MPa) and the required amount of mefenamic acid solution in DMSO-d_6_ for recording NOESY spectra was based on the data of works [85,95,96].

## 4. Conclusions

In this research, the physical and chemical properties of aerogel composite material with mefenamic acid were studied using techniques such as solid-state MAS NMR, high-pressure ^13^C NMR, T_1_–T_2_ RRCOSY, and NOESY. The results show that the presence of mefenamic acid affects the chemical environment of the aerogel, and this change can be seen in the integral intensities of the 2D RRCOSY spectra. Furthermore, ^13^C NMR spectroscopy is a suitable method for studying the kinetics of CO_2_ sorption and can be used to identify the species involved in the reaction. The NOESY study also found a difference in the populations of conformers of mefenamic acid released from the aerogel compared to bulk mefenamic acid. Hence, the ratio of conformers changed from 22% to 78% for doped aerogel to 75% to 25% for a bulk solution. Overall, these results provide insight into the physicochemical properties of the aerogel composite material with mefenamic acid and can be helpful for future drug delivery applications.

## Data Availability

Not applicable.

## Supplementary Materials

The supporting information can be downloaded at: https://www.mdpi.com/article/10.3390/ijms24086882/s1.
