# Supplementary material for: Exploring the Conformational Equilibrium of Mefenamic Acid Released from Silica Aerogels via NMR Analysis
Source: Int J Mol Sci. 2023 Apr 7;24(8):6882. doi: 10.3390/ijms24086882 (PMC10138679; doi:10.3390/ijms24086882)
Supplement: Supplementary file 1 [file ijms-24-06882-s001.zip › ijms-2302269-supplementary.pdf]

Supplementary Materials

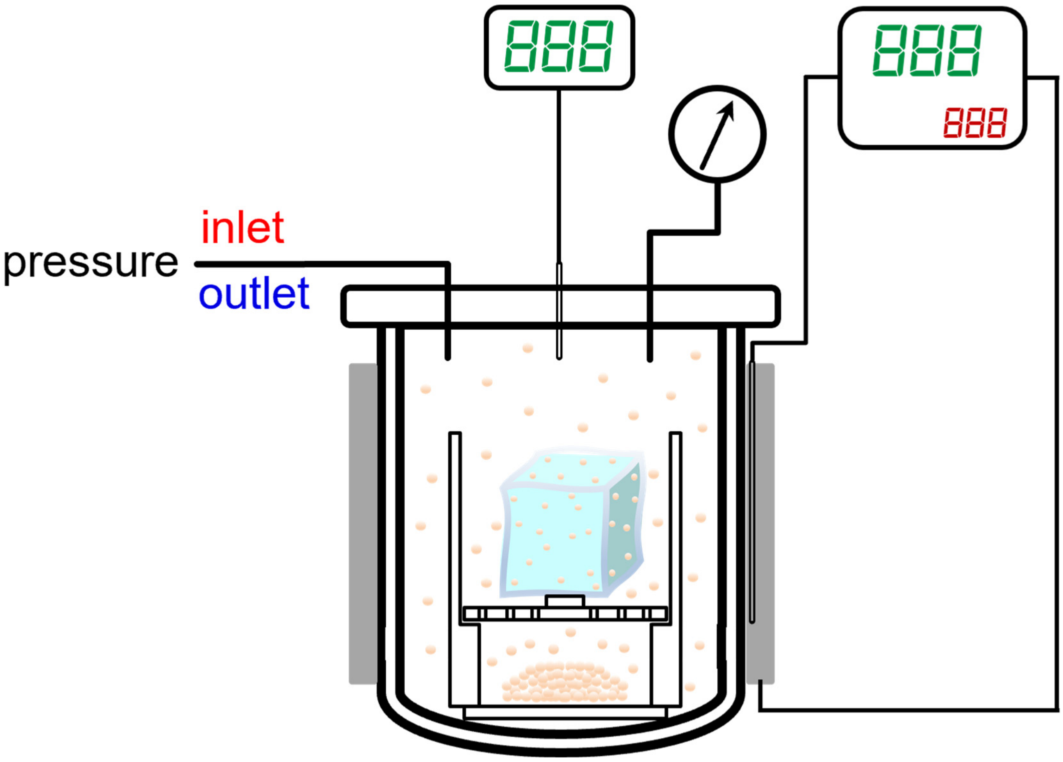

Figure S1. A schematic diagram of an autoclave with a system separating the initial components for impregnating MFA into aerogels.

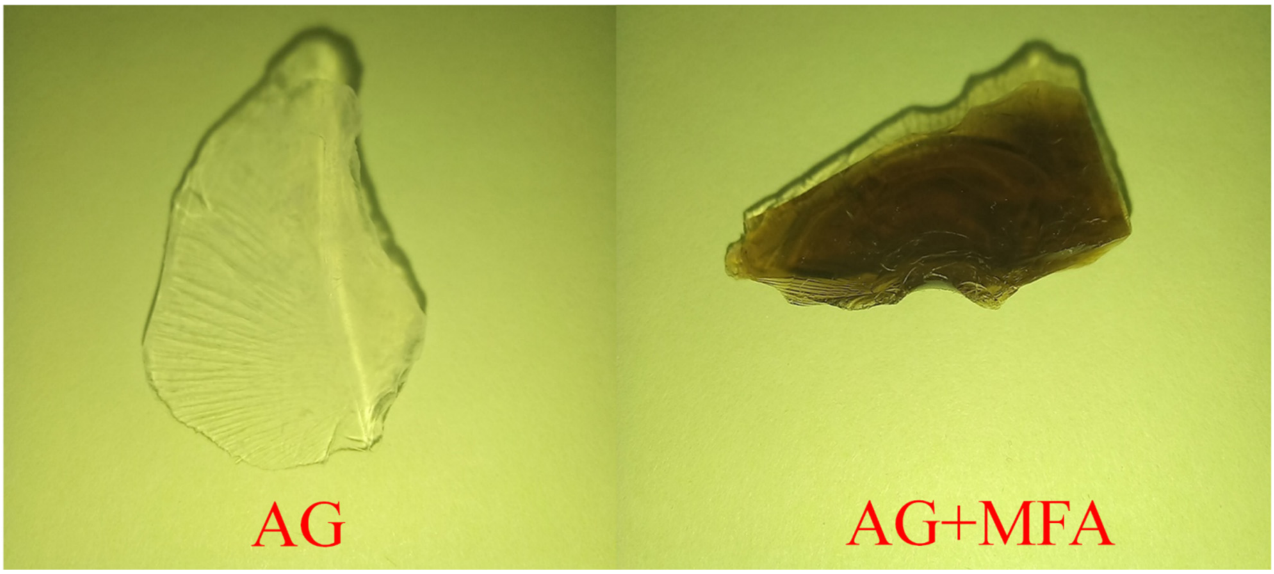

Figure S2. Samples of pure aerogel (transparent) and aerogel impregnated with mefenamic acid (dark yellow color).

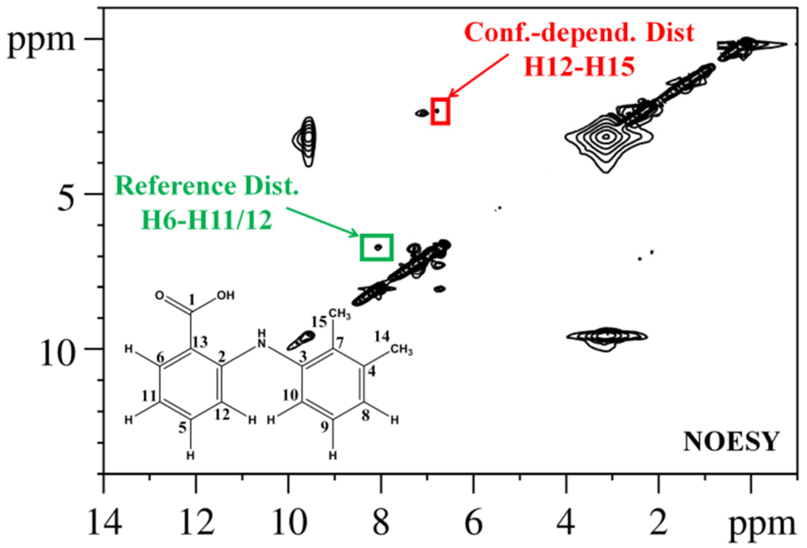

Figure S3. ^{1}H-^{1}H NOESY spectra of mefenamic acid in $scCO_2$ + DMSO-d6 recorded in the presence of an aerogel doped with mefenamic acid at 45 °C and 9 MPa.

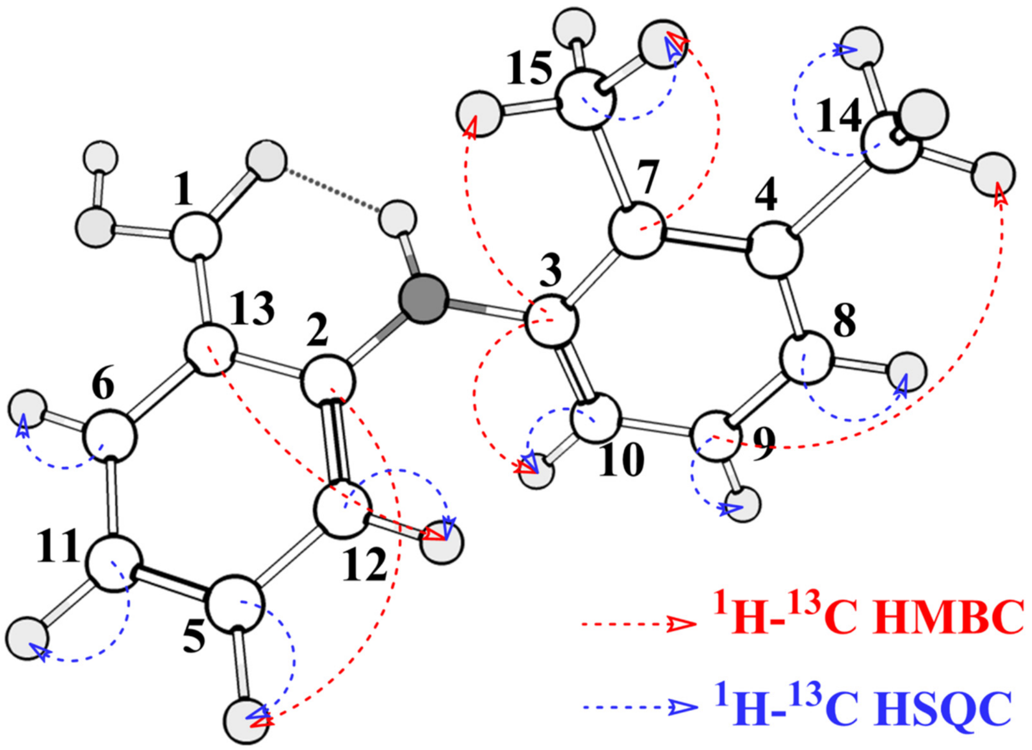

Figure S4. The structure of the mefenamic acid molecule with atom numbering. Red arrows show the intramolecular interactions detected by the ^{1}H-^{13}C HMBC method between hydrogen and carbon atoms separated by several chemical bonds. In contrast, blue arrows indicate the intramolecular interactions detected by the ^{1}H-^{13}C HSQC method between hydrogen and carbon atoms separated by a single chemical bond.

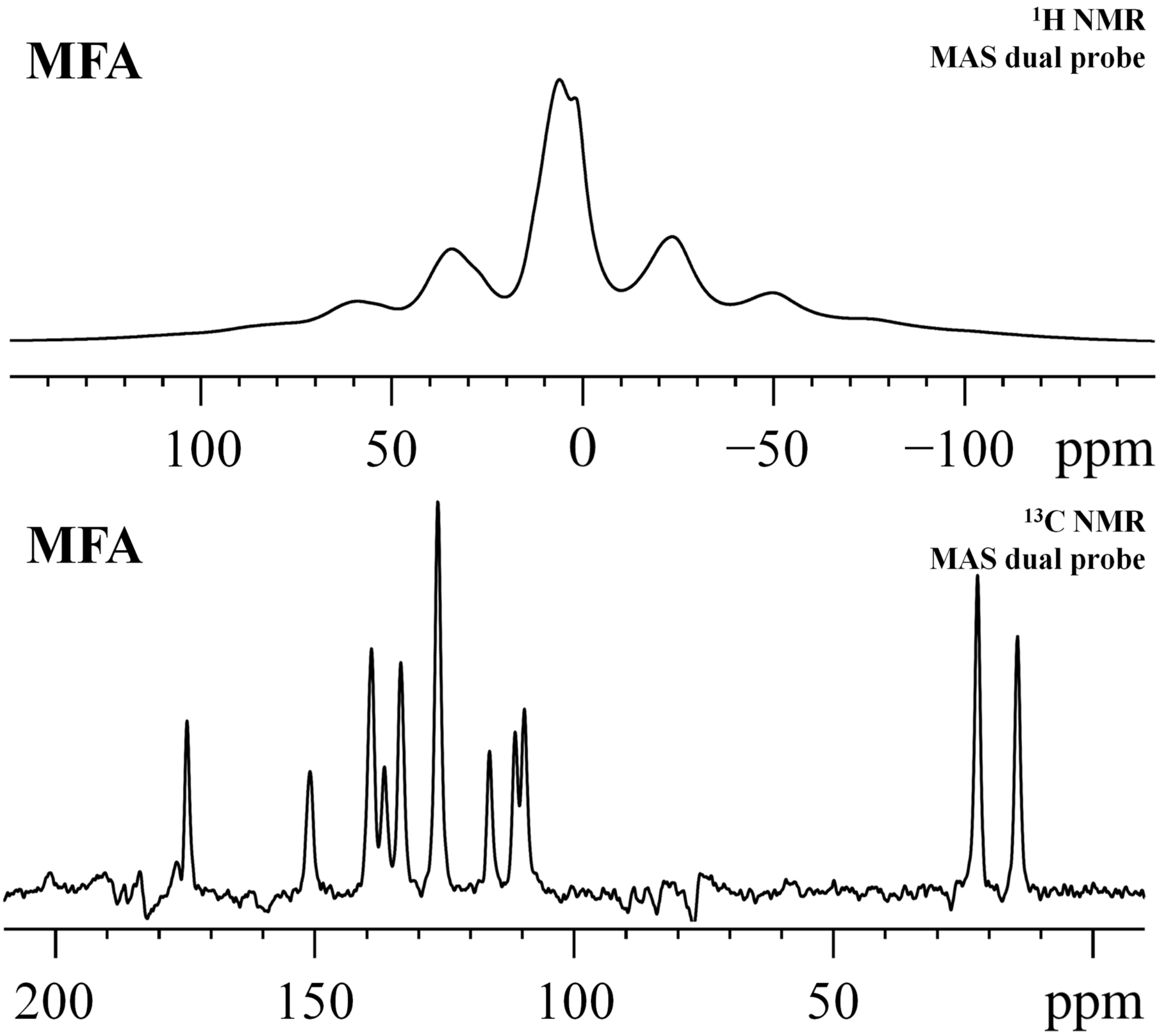

Figure S5. ^{13}C (**top**) and ^{1}H (**bottom**) NMR MAS spectra of mefenamic acid.
